# Dynamic Metabolic Changes During Postmortem Aging of Yili Horsemeat Revealed by Untargeted Metabolomics

**DOI:** 10.3390/ani16030508

**Published:** 2026-02-05

**Authors:** Xixi Yang, Tongliang Wang, Jingtao Gan, Chen Meng, Jingxuan Shen, Zexu Li, Xueyan Li, Yaqi Zeng, Wanlu Ren, Xinkui Yao, Jun Meng

**Affiliations:** 1 College of Animal Science, Xinjiang Agricultural University, Urumqi 830052, China; xxyang2022@126.com (X.Y.); wtl13639911402@163.com (T.W.); ganjintao2022@126.com (J.G.); chenmeng0330@126.com (C.M.); 18914889860@163.com (J.S.); 13593312012@163.com (Z.L.); lixxuey0421@163.com (X.L.); zengyaqi@xjau.edu.cn (Y.Z.); renwanlu@xjau.edu.cn (W.R.); yaoxinkui@xjau.edu.cn (X.Y.); 2 Xinjiang Key Laboratory of Horse Breeding and Exercise Physiology, Urumqi 830052, China; 3 Horse Industry Research Institute, Xinjiang Agricultural University, Urumqi 830052, China

**Keywords:** metabolomics, postmortem aging, Yili horse, muscle fiber

## Abstract

Postmortem aging is a crucial process for improving meat quality; however, the metabolic mechanisms underlying the postmortem maturation of Yili horsemeat remain unclear. In this study, untargeted metabolomics was employed to investigate the dynamic changes in metabolites during postmortem aging of Yili horse longissimus dorsi muscle. Samples from six Yili horses were subjected to aging at 4 °C for 0, 14, and 28 days. Intramuscular Environment and metabolic profiles were analyzed using UHPLC-QTOF-MS. Results showed that meat pH initially decreased and then increased over the aging period (*p* < 0.05), whereas muscle fiber diameter and cross-sectional area gradually declined (*p* < 0.05). Metabolomic analysis revealed significant differences in metabolic profiles among different aging stages. KEGG pathway enrichment analysis indicated that the differentially expressed metabolites (DEMs) were predominantly involved in purine metabolism, nucleotide metabolism, and amino acid biosynthesis pathways. Notably, the degradation of AMP and IMP into inosine and hypoxanthine and the accumulation of free amino acids such as tryptophan and phenylalanine were closely associated with the development of umami taste and overall flavor of horsemeat. These findings elucidate the key metabolic pathways regulating postmortem meat quality in Yili horses and provide a theoretical basis for its production and application.

## Simple Summary

This study explored metabolic changes in Yili horse longissimus dorsi muscle during 0, 14, and 28 days of postmortem aging at 4 °C via untargeted metabolomics. Results showed pH first decreased then increased, while muscle fiber diameter and area declined. Metabolomic analysis identified distinct metabolic profiles across stages, with differential metabolites enriched in purine metabolism, nucleotide metabolism, and amino acid biosynthesis. AMP/IMP degradation into inosine/hypoxanthine and accumulation of tryptophan/phenylalanine enhanced umami and flavor. The findings clarify key metabolic pathways regulating Yili horsemeat quality postmortem, providing a theoretical basis for production and application.

## 1. Introduction

In recent years, as consumers’ lifestyles have evolved and awareness of healthy dietary concepts has grown, their demands and preferences for meat products have also shifted. Horse meat, as a distinctive livestock product, exhibits clear regional patterns in terms of processing and consumption within China. It is primarily concentrated in ethnic minority regions such as Xinjiang and Inner Mongolia [1]. Horsemeat possesses unique nutritional properties; compared with beef (5.7%), donkey meat (5.0%), mutton (7.0%), and pork (6.6%), it has lower fat content, approximately 2.7%, which is rich in high-quality proteins and ideal amino acid profiles [2,3,4]. Horsemeat contains a relatively high glycogen content, which imparts a natural sweetness [5]. Moreover, horsemeat is abundant in vitamins and minerals, including vitamins B and E, calcium, phosphorus, and iron. It also has a relatively high collagen content, which undergoes extended softening and physicochemical transformation under acidic conditions. A distinctive feature of horse muscle fibers is their high proportion of oxidative type I fibers, adapted to the demands of prolonged physical activity [6], which may lead to postmortem metabolic patterns that differ significantly from those of ruminants or monogastric animals. Postmortem aging is a critical processing technique in the meat industry. By regulating a series of biochemical reactions within muscle tissue such as the hydrolysis of structural proteins (myofibrillar and cytoskeletal proteins), lipid oxidation, and transformation of low-molecular-weight metabolites, meat quality can be markedly improved [7]. During this process, endogenous enzymes and microbial activity act synergistically to break down muscle components into flavor precursors and bioactive compounds, enhancing tenderness, juiciness, and flavor complexity [8,9]. Aging also influences meat color and pH. This technique has been widely applied in processing common meats such as beef, mutton, and pork, with numerous studies elucidating the mechanisms by which aging regulates meat quality. Dashdorj et al. reported that prolonged aging enhances beef flavor, primarily due to the release of reducing sugars, free amino acids, and peptides, as well as the breakdown of nucleotides during postmortem aging, generating Inosine 5′-monophosphate (IMP), guanosine monophosphate (GMP), inosine, and hypoxanthine [10].

Untargeted metabolomics, as a comprehensive approach for analyzing all small-molecule metabolites in biological samples, allows for the profiling of metabolites and metabolic patterns, thereby providing insights into changes in meat quality. It has now become an important tool for investigating the mechanisms of postmortem aging in animals [11]. Hernandez et al. employed untargeted metabolomics to analyze beef tenderness and flavor, identifying biomarkers such as carnosine, anserine, and IMP that were significantly associated with these traits, and found that carbohydrate and biogenic amine contents exhibited the most pronounced changes over aging time [12]. Chen et al. studied superchilling treatment (−3 °C) and observed that it accelerated the degradation of μ-calpain and caspase-3, promoted the accumulation of IMP and 4-hydroxyproline, and consequently improved meat tenderness [13]. In recent years, untargeted metabolomics has been widely applied to investigate muscle metabolism and meat quality, enabling the characterization of metabolites and the elucidation of flavor formation mechanisms during meat processing. Research strategies based on metabolomics have been extensively used in beef, lamb, and other meats to identify key biomarkers and core pathways involved in aging. However, studies on postmortem aging in horsemeat remain scarce. Considering the structural and nutritional particularities of horsemeat, its postmortem aging process, including the dynamics of metabolite transformation, activation patterns of key metabolic pathways, and accumulation of flavor precursors, may differ from those of other meats. Therefore, in this study, UHPLC-Q-TOF MS-based untargeted metabolomics was employed to characterize the metabolic profile of Yili horsemeat during postmortem aging, identify key differentially expressed metabolites and enriched pathways, and reveal the dynamic changes in metabolites. These findings provide a theoretical basis for the application of postmortem aging in horsemeat production.

## 2. Materials and Methods

### 2.1. Experimental Animals

**Figure 1:**
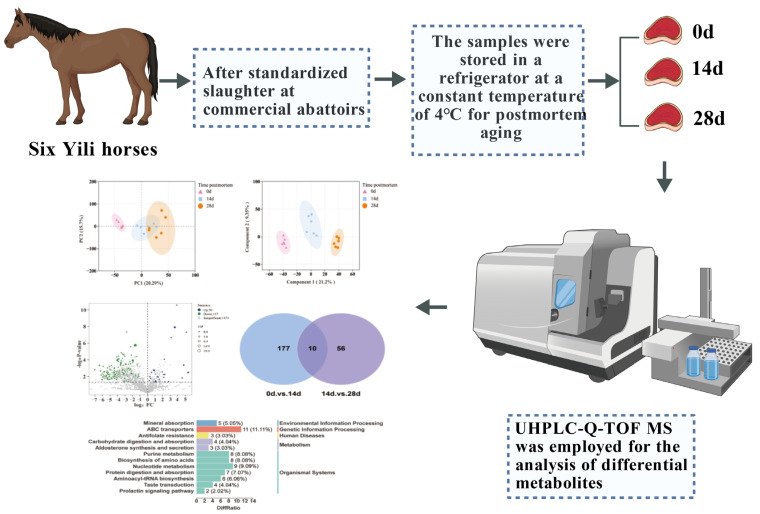
Technical flowchart of the study on different postmortem aging periods of Yili horsemeat.

Six male Yili horses (aged 24–36 months, body weight of 295 ± 20 kg) were selected as biological replicates (n = 6). The horses were transported to a slaughterhouse and allowed to rest in the stables for 10 h with fasting and water deprivation prior to slaughter (In accordance with Welfare criteria for animals to be slaughtered: GB/T 42304-2023) [14]. Slaughter was performed by exsanguination, followed by removal of the hide. One hour postmortem, longissimus dorsi muscle samples were collected from each carcass (n = 6), divided into three equal portions, placed in polyethylene bags, and stored at 4 °C for postmortem aging at 0, 14, and 28 days (Figure 1). At each time point, longissimus dorsi muscle samples from six Yili horses were separately collected for pH measurement, histological observation, enzyme activity assay, and metabolomics analysis.

### 2.2. Analysis of Muscle pH Value

The muscle pH values at 0, 14, and 28 days postmortem were measured using a pH-STAR portable pH meter equipped with a muscle-specific puncture probe. The calibration procedure was as follows: a three-point calibration was performed using standard buffers at pH 4.01, 7.00, and 9.21. Sample measurements were conducted immediately after calibration. For each of muscle samples (n = 6), pH was measured three times at each aging stage (0, 14, 28 days), and the mean value of the three replicates per sample was calculated. The final reported values represent the average of these six sample-specific means ± SD.

### 2.3. Histological Characteristics of Muscle

Muscle tissue samples were collected from Yili horses at 0, 14, and 28 days postmortem and fixed in paraformaldehyde solution. The samples were then rinsed, dehydrated, embedded, sectioned, and stained with hematoxylin–eosin. After mounting on resin slides, muscle fiber structures at different postmortem aging stages were observed and photographed using a Motic digital slide scanner. For each individual, five random fields per section were selected, and ten muscle fibers per field were measured for fiber diameter and cross-sectional area using Image Pro Plus 6.0 software. Data were expressed as mean ± SD and analyzed by one-way ANOVA.

### 2.4. Analysis of Metabolic Enzyme Activity and Myoglobin Content

Muscle samples (0.5 g) from 0, 14, and 28 days postmortem were homogenized with PBS using a glass homogenizer on ice. The homogenates were centrifuged at 5000× *g* for 10 min, and the supernatants were collected. Concentrations of oxy-myoglobin (MbO_2_), deoxy-myoglobin (DeOxyMb), and metmyoglobin (MetMb) were determined using corresponding ELISA kits, with absorbance recorded at 540 nm. Lactate dehydrogenase (LDH) and pyruvate kinase (PK) activities were measured using commercial kits according to the manufacturer’s instructions.

### 2.5. UHPLC-Q-TOF MS Metabolomics Analysis

Differential metabolite analysis of muscle tissues at 0, 14, and 28 days postmortem was performed using ultra-high-performance liquid chromatography coupled with tandem mass spectrometry (UHPLC-MS/MS).

#### 2.5.1. Metabolite Extraction

Samples were slowly thawed at 4 °C, and an appropriate amount of tissue was added to pre-cooled methanol/acetonitrile/water solution (2:2:1, *v*/*v*), followed by vortex mixing. The mixture was subjected to low-temperature ultrasonication for 30 min, left at −20 °C for 10 min, and centrifuged at 14,000× *g* and 4 °C for 20 min. The supernatant was vacuum-dried and reconstituted with 100 μL acetonitrile/water solution (acetonitrile/water = 1:1, *v*/*v*) prior to mass spectrometry. The reconstituted solution was vortexed and centrifuged at 14,000× *g* and 4 °C for 15 min, and the supernatant was injected for analysis.

#### 2.5.2. UHPLC-Q-TOF MS Analysis

Samples were subjected to gradient elution in an autosampler maintained at 4 °C. Mass spectrometry analysis was performed using a Triple TOF 6600 instrument in both positive and negative electrospray ionization (ESI) modes. Source parameters were set as follows: Gas1 = 60, Gas2 = 60, CUR = 30 psi, ion source temperature = 600 °C. The primary mass-to-charge (*m*/*z*) detection range was 60–1000 Da, and the secondary range was 25–1000 Da. Accumulation time per scan was 0.20 s/spectrum for MS1 and 0.05 s/spectrum for MS2. Analysis was performed in IDA mode with DP ± 60 V and collision energy of 35 ± 15 eV. Orbitrap Exploris™ 480 analysis was performed in ESI positive and negative ion modes. Source parameters were Gas1 = 50, Gas2 = 2, ion source temperature = 350 °C, ISVF 3500 V for positive mode and 2800 V for negative mode. The primary *m*/*z* detection range was 70–1200 Da with a resolution of 60,000 and a scan accumulation time of 100 ms. MS2 data were acquired in segmented mode with the same *m*/*z* range and resolution, scan accumulation time 100 ms, and dynamic exclusion time of 4 s.

#### 2.5.3. Bioinformatics Analysis

Metabolomics data were imported into MetaboAnalyst v. 4.0 for principal component analysis (PCA), with all variables containing missing values removed. Data were normalized using the quantile method. To identify key metabolites across the three postmortem aging stages, *t*-tests were performed. Orthogonal projections to latent structures discriminant analysis was conducted using SIMCA 14.1 software. Features satisfying both *p* < 0.05 and VIP > 1 were considered potential key metabolites.

Pathway analysis of differential metabolites was performed using the Kyoto Encyclopedia of Genes and Genomes (KEGG) database, and Fisher’s exact test was applied to identify the most relevant biological pathways. *p*-values corrected by the Benjamini–Hochberg method (<0.05) were considered statistically significant. Normalized data were used to generate clustered heatmaps. Weighted gene co-expression network analysis (WGCNA) was performed using the WGCNA package in R to construct co-expression networks. Phenotypic data were incorporated into the analysis to evaluate correlations between phenotypes and metabolite modules and to predict potential functional relationships.

### 2.6. Statistical Analysis

Enzyme activity data were analyzed by one-way analysis of variance (ANOVA) using IBM SPSS 26.0 software. Data are presented as mean ± standard deviation (SD), and differences were considered statistically significant at *p* < 0.05.

## 3. Results

### 3.1. Changes in pH and LDH and PK Activities of Yili Horsemeat During Postmortem Aging

**Figure 2:**
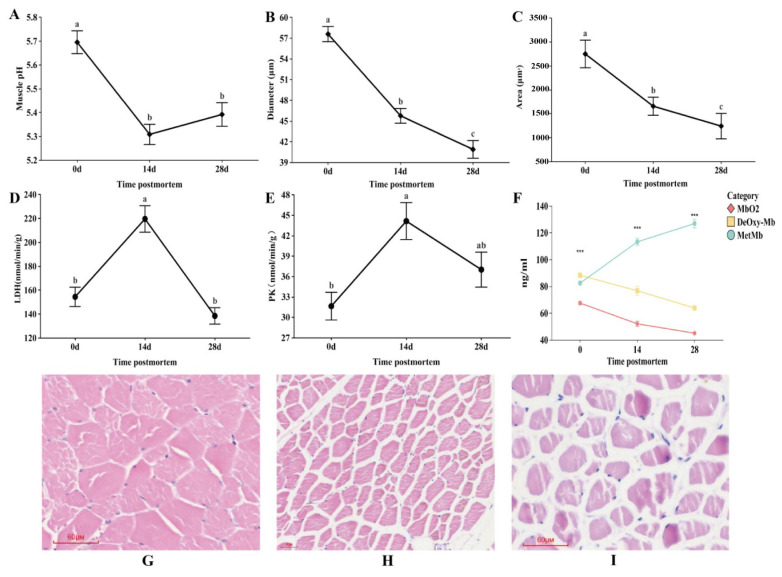
Changes in the muscle internal environment and tissue structure during the postmortem aging of Yili horsemeat. (**A**) pH value; (**B**) Measured diameter of muscle fibers; (**C**) Measured area of muscle fibers; (**D**) LDH activity; (**E**) PK activity; (**F**) Changes in myoglobin content; (**G**) Muscle fiber tissue sections at 0 d (20×); (**H**) Muscle fiber tissue sections at 14 d (10×); (**I**) Muscle fiber tissue sections at 28 d (20×). Lowercase letters indicate significant differences during postmortem aging (*p* < 0.05). ***: indicates a highly significant difference (*p* < 0.05).

During storage, changes in meat pH may affect the activity of related enzymes and the electron transport chain of the metmyoglobin-reducing system, thereby indirectly influencing meat color stability. The pH changes in longissimus dorsi muscle of Yili horses at different postmortem aging stages at 4 °C are shown in Figure 2A. The highest pH value (5.69) was measured immediately postmortem (0 days). At 14 days postmortem, pH significantly decreased, reaching a minimum of 5.30 (*p* < 0.05). During the period from 14 to 28 days postmortem, pH slightly increased to 5.38, with no significant change observed (*p* > 0.05). With the extension of postmortem aging time, LDH and PK activities exhibited an initial increase followed by a decrease (Figure 2D,E). Immediately postmortem, LDH and PK activities were low. At 14 days, both reached their maximum levels. From 14 days onward, LDH activity decreased significantly (*p* < 0.05), reaching its minimum at 28 days, while the decrease in PK activity was slower than that of LDH.

### 3.2. Muscle Tissue Structure Observations at Different Postmortem Aging Stages

Significant changes in the structure of longissimus dorsi muscle were observed at different postmortem aging stages in Yili horses (Figure 2B,C). At 0 days postmortem, myofibers were closely arranged, the intercellular spaces were small, cell boundaries were clear, and myofibers were plump and continuous (*p* < 0.05) (Figure 2G). As aging progressed to 14 days, muscle cells began to contract, and the cross-sectional area and diameter of myofibers gradually decreased (*p* < 0.05) (Figure 2H). The spaces between myofibers widened, and some cells showed slight fragmentation. At 28 days, the inter-fiber gaps further increased, myofiber area and diameter reached their minimum values (Figure 2I), and some regions showed fiber breakage and slight matrix dissolution, indicating enhanced protein degradation, which is favorable for meat tenderization. These structural changes were consistent with the pH fluctuation trend, suggesting that the acidic environment during aging promoted the hydrolysis of myofibrillar proteins.

### 3.3. Changes in Myoglobin Content at Different Postmortem Aging Stages

Myoglobin in muscle is a protein specialized for storing oxygen to provide energy for muscle contraction. In muscle tissue, myoglobin is mainly in the deoxygenated form (DeOxy-Mb), which forms oxy-myoglobin (MbO_2_) upon exposure to O_2_. Figure 2F shows the changes in three forms of myoglobin DeOxy-Mb, MbO_2_, and MetMb in Yili horse muscle at different postmortem aging stages. DeOxy-Mb and MbO_2_ decreased with increasing aging time, whereas MetMb increased over time, indicating that oxygen content in muscle cells of Yili horsemeat gradually decreased as aging progressed.

### 3.4. Metabolomics Analysis

#### 3.4.1. Multivariate Analysis

**Figure 3:**
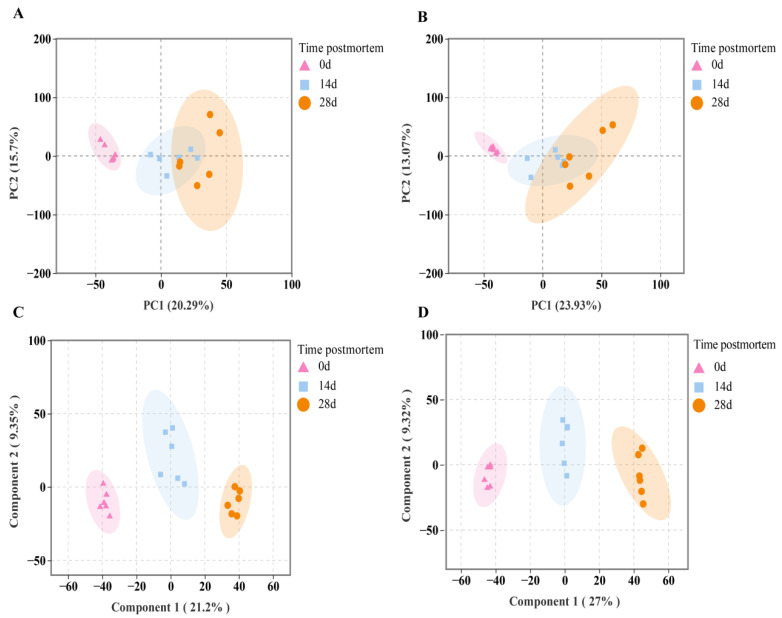
Multivariate statistical analysis of identified metabolites during postmortem aging. (**A**) PCA score plots of samples in positive ion mode; (**B**) PCA score plots of samples in negative ion mode; (**C**) OPLS-DA score plots of samples in positive ion mode; (**D**) OPLS-DA score plots of samples in negative ion mode.

During postmortem aging, ultra-high-performance liquid chromatography coupled with quadrupole time-of-flight mass spectrometry (UHPLC-Q-TOF MS) detected 9972 peaks in muscle samples at different time points, including 4885 positive ion peaks and 5087 negative ion peaks. From 0 to 14 days postmortem, a total of 1661 metabolites were identified, with 879 in positive ion mode and 793 in negative ion mode. In the 14–28 day comparison group, a total of 1661 metabolites were identified, including 908 in positive ion mode and 753 in negative ion mode. To further distinguish the differential mass features of metabolites across postmortem aging stages, positive and negative ion modes were analyzed using a combination of PCA and orthogonal partial least squares discriminant analysis to ensure data accuracy. PCA analysis indicated that all samples from the three postmortem aging stages fell within the 95% confidence interval. As aging time increased, the 0-day samples separated from the other two aging stages, indicating distinct metabolic patterns at 0 days compared to the other stages. PCA results for 14 and 28 days showed clustering with partial overlap, suggesting similarity between these two stages (Figure 3A). Similarly, PCA results in negative ion mode were consistent with those in positive ion mode (Figure 3B).

PCA is an unsupervised model, whereas OPLS-DA is a supervised model, which can further distinguish specific metabolites and improve model reliability. OPLS-DA score plots were constructed for the three postmortem aging stages in both positive and negative ion modes. As shown in Figure 3C,D, clear separation was observed between groups in both ion modes, indicating that the OPLS-DA model is reliable. Therefore, metabolite profiles differed among Yili horse muscle samples at different postmortem aging times.

#### 3.4.2. Identification of Metabolites at Different Postmortem Aging Stages

**Figure 4:**
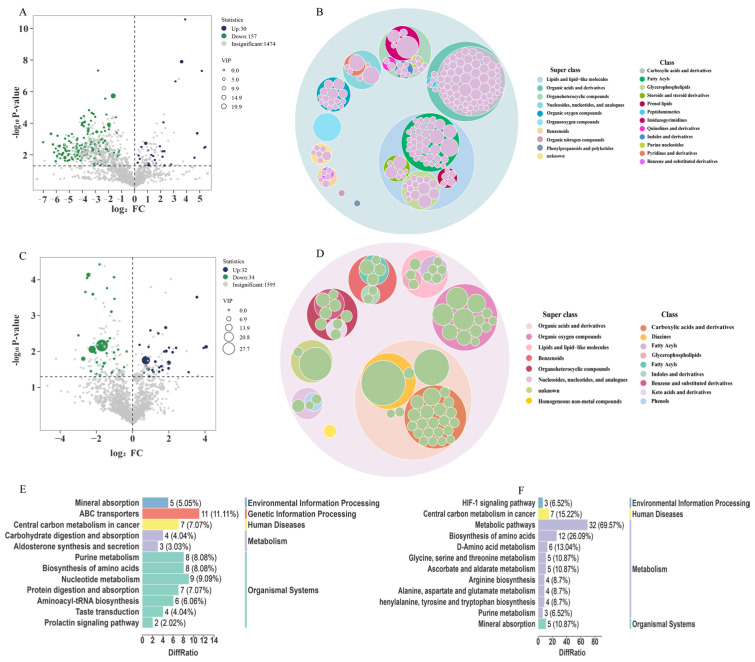
Identification and enrichment analysis of metabolites during postmortem aging. (**A**) 0 d vs. 14 d DEMs (volcano plot); (**B**) 0 d vs. 14 d DEMs (primary and secondary classification); (**C**) 14 d vs. 28 d DEMs (volcano plot); (**D**) 14 d vs. 28 d DEMs (primary and secondary classification); (**E**) KEGG enrichment of 0 d vs. 14 d DEMs; (**F**) KEGG enrichment of 14 d vs. 28 d DEMs.

Differential metabolites at different postmortem aging stages were identified from the OPLS-DA model using the criteria VIP > 1 and *p* < 0.05. In the 0–14 day comparison group, a total of 187 differential metabolites were obtained, including 30 upregulated and 157 downregulated metabolites (Figure 4A, Table S1). In the 14–28 day comparison group, 66 differential metabolites were detected, of which 32 were upregulated and 34 were downregulated (Figure 4C, Table S2).

The differential metabolites identified at different postmortem aging stages were classified into primary and secondary categories. The results showed that in the 0–14 days group, the metabolites were classified into 10 SuperClasses, predominantly including Organic acids and derivatives (71 species, 38%), Lipids and lipid-like molecules (64 species, 34.2%), Organoheterocyclic compounds (18 species, 9.6%), Organic oxygen compounds (14 species, 7.5%), unknown (6 species, 3.2%), Nucleosides, nucleotides, and analogs (6 species, 3.2%), Benzenoids (5 species, 2.7%), Organic nitrogen compounds (1 species, 0.5%), Organooxygen compounds (1 species, 0.5%), and Phenylpropanoids and polyketides (1 species, 0.5%) (Figure 4B). In the 14–28 days group, the metabolites were categorized into 8 SuperClasses, with Organic acids and derivatives being the most abundant (14 species, 37.61%), followed by Organic oxygen compounds (12 species, 18.2%), Lipids and lipid-like molecules (8 species, 12.1%), Organoheterocyclic compounds (8 species, 12.1%), Benzenoids (6 species, 9.1%), Nucleosides, nucleotides, and analogues (3 species, 4.5%), Homogeneous non-metal compounds (1 species, 1.5%), and unknown (2 species, 3%) (Figure 4D).

To further determine unique and shared metabolites between the two comparison groups, Venn diagram analysis was performed (Figure S1). The results showed that 177 metabolites were unique to the 0–14 day group, and 56 metabolites were unique to the 14–28 day group. The greatest number of differential metabolites was observed between 0 and 14 days, whereas the least difference occurred between 14 and 28 days. Ten differential metabolites were shared among 0, 14, and 28 days. These results indicate that the metabolic profiles of Yili horse muscle vary significantly at different postmortem aging times.

#### 3.4.3. Enriched Differential Metabolic Pathways at Different Postmortem Aging Stages

To further investigate changes in metabolic pathways during postmortem aging of Yili horse muscle, differential metabolites were analyzed using the KEGG database, and differential metabolic maps were generated for the two comparison groups. Significant KEGG pathways identified in the 0–14 day group are listed in Table 1. The differential metabolites in the 0–14 days comparison group were mainly enriched in signaling pathways such as Mineral absorption, Nucleotide metabolism, Purine metabolism, Biosynthesis of amino acids, Central carbon metabolism in cancer, and Taste transduction (Figure 4E). In contrast, the differential metabolites in the 14–28 days comparison group were primarily enriched in Metabolic pathways, Biosynthesis of amino acids, Central carbon metabolism in cancer, HIF-1 signaling pathway, and Purine metabolism (Figure 4F). The identified major metabolites are shown in Table 1, and a heatmap was generated to visualize the dynamic changes in the main metabolites (Figure S2), indicating that these metabolites may influence meat quality at different aging times.

**Figure 5:**
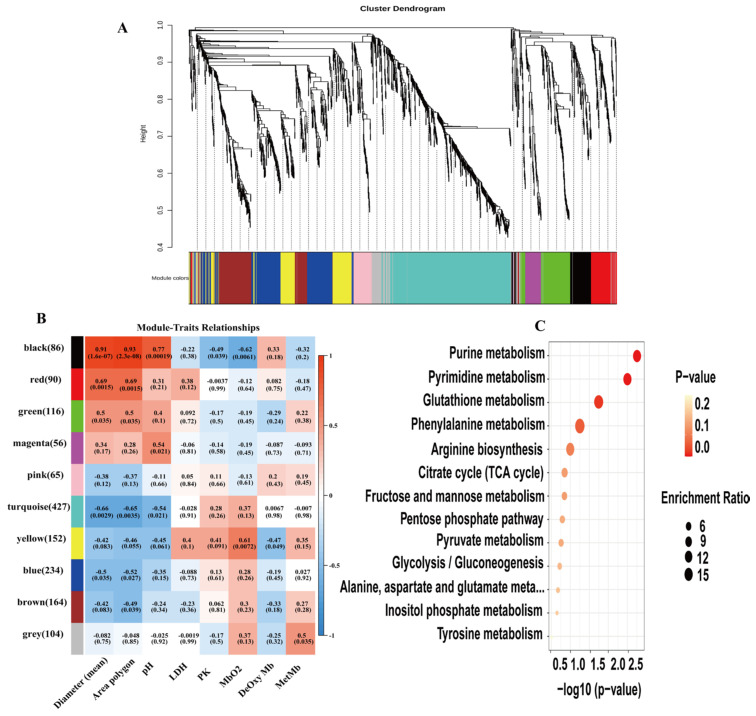
Weighted gene co-expression network analysis (WGCNA) of differentially expressed metabolites (DEMs) during three postmortem aging periods. (**A**) Dendrogram and modules of all DEMs; (**B**) Correlation between module eigengenes (MEs) and sample traits (color bar on the right indicates the correlation range from negative to positive); (**C**) KEGG pathways enriched with DEMs in the black module.

To further study potential key metabolites associated with mean fiber diameter, fiber area (polygon), pH, and LDH across three postmortem aging stages, WGCNA was performed on the DEMs. DEMs were grouped into 10 modules, with module sizes ranging from 65 to 427 metabolites (Figure 5). Correlation analysis between module eigengenes and fiber diameter (mean), fiber area (polygon), and pH showed that the black module was positively correlated with diameter (r = 0.91, *p* < 0.00), area (r = 0.93, *p* < 0.00) and pH (r = 0.77, *p* < 0.00019), while negatively correlated with MbO_2_ (r = −0.62, *p* < 0.0061). The red module was positively correlated with diameter and area. Among them, the black module showed the strongest correlations. Metabolites in the black module were mainly enriched in purine metabolism (Table S3), consistent with the pathways identified by KEGG enrichment analysis.

**Figure 6:**
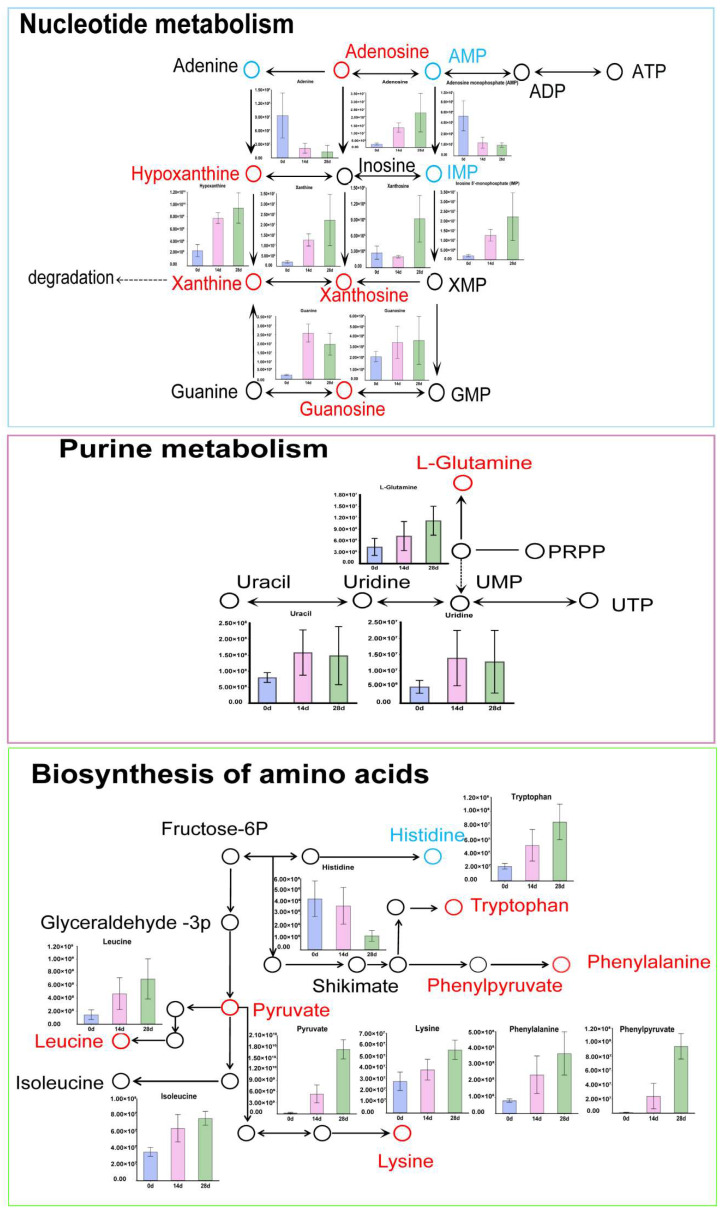
Common pathways from KEGG enrichment analysis in postmortem aged horsemeat. Red circles indicate upregulated metabolites, blue circles represent downregulated metabolites, and black circles denote metabolites not detected in the samples.

KEGG enrichment analysis revealed three common pathways between the two comparison groups, namely Purine metabolism, Biosynthesis of amino acids, and Nucleotide metabolism. Analysis of these pathways showed that in nucleotide metabolism, metabolites such as adenosine, hypoxanthine, xanthine, and guanosine gradually increased with aging time, whereas Adenine, AMP, and IMP decreased over time. In the purine metabolism pathway, L-glutamine gradually increased during postmortem aging. In the biosynthesis of amino acids pathway, tryptophan, phenylalanine, leucine, pyruvate, and lysine showed an increasing trend during aging, whereas histidine decreased over time (Figure 6).

## 4. Discussion

Postmortem aging is a critical stage in the conversion of muscle to meat. After slaughter, the muscle environment changes, leading to alterations in the tissue microenvironment and a gradual shift from aerobic to anaerobic metabolism. This process involves a series of complex biochemical changes, including Glycolysis, oxidative phosphorylation, and phosphocreatine metabolism, which ultimately affect meat quality [15].

pH is an important indicator for evaluating meat quality and plays a key role in changes in meat tenderness. The extent of glycolysis and accumulation of lactic acid before and after slaughter largely determine the ultimate pH of meat [16]. Li et al. reported that during postmortem aging, the pH of Qinchuan beef initially decreased and then increased, reaching a minimum on day 4 [17]. Studies have shown that pH changes dynamically during beef aging: in the early postmortem period, lactic acid produced by glycolysis causes the pH to rapidly drop to approximately 5.5. As aging progresses, metabolic activities such as mitochondrial respiration and phosphocreatine degradation gradually decline, leading to a slow increase in pH to 5.8–6.0, a dynamic process that contributes to improved tenderness [18]. Wang et al. used kinetic modeling to analyze postmortem beef and found that early postmortem, ATP production is dominated by glycolysis, causing a rapid pH decline. As glycogen is depleted, phosphocreatine degradation and mitochondrial aerobic respiration (albeit weak) gradually become secondary ATP sources, leading to a slow pH recovery, indicating that energy metabolism regulates pH changes during beef aging [19]. In this study, the pH of Yili horse muscle gradually decreased from 0 to 14 days postmortem, reaching the lowest value, and then showed a slow increase after 14 days, consistent with previous studies. The early postmortem pH decline in horsemeat was mainly due to the shift in metabolic pathways, resulting in lactic acid accumulation. As aging progressed, pH began to rise slowly, as protein and amino acid degradation may produce alkaline substances such as ammonia, which neutralize lactic acid and lead to a gradual pH increase [20]. LDH and PK play key roles in metabolic regulation during postmortem aging. As key enzymes in glycolysis, they are decisive in energy metabolism, pH changes, and the development of meat quality traits [21]. PK, as a rate-limiting enzyme in glycolysis, is rapidly activated in the early postmortem period to accelerate the conversion of phosphoenolpyruvate (PEP) to pyruvate, thereby rapidly generating ATP to maintain basic cellular functions [22]. Studies have shown that during prolonged beef aging, LDH and PK activities increase in the early stage due to accelerated glycolysis, lactic acid accumulation, and ATP demand, and decrease in the later stage as lactic acid levels stabilize. This trend is related to adaptive regulation of glycolytic metabolism [23]. Studies have shown that PK is also involved in regulating the tricarboxylic acid (TCA) cycle, oxidative phosphorylation, and the phosphorylation state of myofibrillar proteins, thereby indirectly affecting muscle contractile properties and postmortem tenderization [24]. Horsemeat due to its high glycogen content, undergoes intense postmortem glycolysis, and rapid lactic acid accumulation leads to a sharp decline in pH [25]. The acidic environment can temporarily enhance the catalytic efficiency of PK, while increased LDH activity can further lower pH [26]. In this study, during the early postmortem period (0–14 days), LDH and PK activities increased over time, whereas from 14 to 28 days, their activities showed a decreasing trend. The trends in LDH and PK activities were inversely correlated with pH changes, consistent with previous studies. Accumulation of lactic acid generated by LDH activity causes pH to drop; when pH decreases to a certain level, LDH activity is inhibited, slowing the reduction of pyruvate to lactate, resulting in pyruvate accumulation and decreased LDH activity [27]. Kaimbayeva et al. reported that in Red Deer meat, myofiber diameter and area both decreased during postmortem aging [28]. Studies have indicated that as postmortem aging progresses, myofiber boundaries become blurred and fibers are loosely arranged. These structural changes are associated with tissue protease degradation, which is activated when pH declines postmortem. These proteases degrade muscle structural proteins, leading to disruption of muscle architecture and loss of structural integrity [29,30]. In this study, during postmortem aging, Yili horse muscle exhibited tightly arranged fibers with small intercellular spaces and clear cell boundaries. As aging progressed, muscle cells contracted, inter-fiber gaps increased, some regions showed fiber breakage, and the matrix was slightly dissolved, while myofiber area and diameter decreased, consistent with previous studies. During postmortem aging, the decline in pH leads to degradation of structural proteins constituting myofibrils, including actin, troponin, myosin, and Z-line proteins, resulting in loosely arranged fibers, enlarged gaps, and blurred boundaries, which reduces myofiber area and diameter. This further indicates enhanced protein degradation, favorable for meat tenderization. Studies have shown that in beef longissimus dorsi and psoas major muscles, MbO_2_ content decreases significantly from 0 to 15 days postmortem, while MetMb continuously increases; this trend is related to mitochondrial activity and the expression of antioxidant enzymes [31], consistent with previous research. In this study, DeOxy-Mb and MbO_2_ decreased with aging time, whereas MetMb increased over time. In the early postmortem period, muscle tissue maintains high metabolic activity, and mitochondria continue to consume residual oxygen, leading to a rapid decline in tissue oxygen partial pressure [32], which promotes the conversion of MbO_2_ to DeOxy-Mb. Therefore, at 0 days postmortem, DeOxy-Mb content was higher than MbO_2_ content in this study.

Metabolomics can provide new insights into postmortem aging by comprehensively analyzing small-molecule metabolites, such as amino acids, organic acids, nucleotides, carbohydrates, and volatile compounds, allowing for precise characterization of the entire process by which muscle transitions from living tissue to edible meat [33,34]. During postmortem aging, metabolic processes are not limited to energy metabolism but also involve amino acid and purine metabolism. These multifaceted metabolic changes are crucial for understanding the biochemical transformations occurring at this stage [35]. Based on metabolomic analysis, by comparing differentially expressed metabolites at various aging stages, the dynamic changes in numerous metabolites were revealed, including glycolytic products, compounds related to energy metabolism, purine- and pyrimidine-related compounds, as well as amino acids.

In the early postmortem period, due to the interruption of oxygen supply, muscle cells rapidly shift from aerobic metabolism to anaerobic glycolysis, leading to rapid ATP consumption. Purines are involved in energy supply, metabolic regulation, and coenzyme composition in biological systems. Purine metabolism mainly includes anabolic and catabolic pathways [36]. Studies have shown that during postmortem aging, purine metabolism in beef increases, and to maintain energy homeostasis, muscle cells activate nucleotide degradation pathways, particularly the purine nucleotide cycle [37]. In this study, metabolites related to purine and nucleotide metabolism were present throughout the aging process, and their levels changed dynamically over time. During aging, ATP is degraded via the formation of IMP or inosine to hypoxanthine or xanthine. Research indicates that IMP, inosine, and hypoxanthine play important roles in meat flavor formation, especially in the development of umami taste [38]. In this study, at 0 days postmortem, AMP and IMP had relatively high initial levels. With increasing aging time, by 14 days postmortem, the contents of AMP and IMP decreased, with AMP being degraded to adenosine and adenine, and IMP gradually degraded to inosine and hypoxanthine. Inosine reached its highest content at 14 days, indicating that the umami taste of horsemeat was optimal at this stage. Moreover, postmortem muscle is susceptible to oxidative stress, and L-glutamine is an important precursor for glutathione (GSH) synthesis. As aging progresses, cells increase the utilization and accumulation of glutamine to maintain redox balance and support the antioxidant defense system. These results suggest that the breakdown of metabolites involved in nucleotide and purine metabolism is enriched from 14 to 28 days, contributing to the formation of flavor compounds during aging and potentially serving as an indicator of aging degree. The metabolomic analysis revealed similar changes in the abdominal muscle pathway of horsemeat and donkey meat during postmortem aging, indicating the significant role of purine metabolism in the maturation process of meats from equine species [39].

The biosynthetic metabolic pathways of amino acids play a crucial role in postmortem muscle aging, and the metabolites they contain significantly influence meat quality traits, such as tenderness, flavor development, and overall sensory characteristics [40]. Amino acid metabolites mainly originate from protein degradation. During postmortem aging, endogenous proteases (such as calpains and cathepsins) are activated, leading to the degradation of myofibrillar and cytoplasmic proteins, loosening of the muscle fiber structure, and release of free amino acids. This results in the accumulation of free amino acids (such as glutamate, leucine, and isoleucine) and short peptides [41,42], consistent with the muscle fiber structural changes observed in this study. Through Maillard reactions and Strecker degradation, free amino acids can directly enhance flavor intensity. Free amino acids serve as substrates for the formation of volatile compounds related to aroma, which are important for the development of volatile aromatic substances and meat palatability [43]. Muroya et al. reported that in Japanese Black cattle, glutamate, glutamine, branched-chain amino acids, leucine, and isoleucine exhibited dynamic fluctuations over time [41]. In this study, most amino acid metabolites showed an increasing trend, consistent with previous research. Phenylalanine contributes to umami taste [43]; during postmortem aging of Yili horse muscle, the contents of tryptophan and phenylalanine gradually increased, promoting flavor formation. Furthermore, studies have demonstrated that the accumulation of amino acid metabolites during postmortem aging is associated with the growth of microorganisms such as molds and yeasts [44]. Molds and yeasts release proteases into the meat, increasing the release of free amino acids, thereby improving the tenderness and flavor of horsemeat. These results indicate that as aging progresses, small-molecule nutrients continue to accumulate, promoting the formation of flavor compounds and ultimately enhancing the quality of aged horsemeat.

## 5. Conclusions

In this study, untargeted metabolomics was used to comprehensively investigate the metabolic characteristics of Yili horse muscle during postmortem aging. During aging, significant changes were observed in meat pH, metabolic enzyme activities, muscle tissue structure, and myoglobin content. Specifically, pH showed a decreasing trend followed by a gradual increase, while myofiber diameter and area decreased with aging time, indicating enhanced protein degradation, which is favorable for improving tenderness. Differential metabolites were mainly enriched in pathways such as purine metabolism, nucleotide metabolism, and amino acid biosynthesis. Among these, the degradation of AMP and IMP to inosine and hypoxanthine, as well as the accumulation of free amino acids such as tryptophan and phenylalanine, were closely associated with the development of umami taste and overall flavor in horsemeat. In summary, this study provides metabolomic evidence for understanding the mechanisms underlying meat quality formation in horses and offers a theoretical basis for further research on horsemeat.

## Figures and Tables

**Table 1:** Major differential metabolites identified during postmortem aging.

Group	Metabolite	Fold Change	*p*-Value	VIP
0 d–14 d	21-hydroxyprogesterone	0.02	0.0028	6.4419
	Adenine	4.35	0.0060	2.5666
	Adenosine 5′-diphosphoribose	43.64	0.0032	1.3466
	Corticosterone	0.02	0.0040	1.1484
	D-Fructose	0.27	0.0100	1.3542
	D-galacturonic acid	0.22	0.0000	1.0736
	D-ribose	0.03	0.0013	1.6954
	DL-serine	1.76	0.0449	1.5445
	DL-tryptophan	0.43	0.0068	1.1840
	DL-tyrosine	0.40	0.0136	1.6522
	Guanine	0.10	0.0010	1.2992
	Guanosine 5′-monophosphate(GMP)	8.84	0.0000	1.0952
	His-ser	0.68	0.0451	1.0589
	Hypoxanthine	0.32	0.0000	19.8635
	Inosine	0.26	0.0001	12.4018
	Inosine 5′-monophosphate(IMP)	12.26	0.0000	10.3190
	L-methionine	0.18	0.0010	1.4100
	Lactose	0.01	0.0016	1.4412
	Leucine	0.31	0.0112	3.8721
	Maltose	0.09	0.0003	2.7323
	Maltotriose	0.06	0.0001	3.4224
	O-succinyl-l-homoserine	0.02	0.0056	1.1947
	Phenylalanine	0.34	0.0073	2.7098
	Piperidine	0.24	0.0050	3.5725
	Progesterone	0.06	0.0040	1.3692
	Sedoheptulose 7-phosphate	0.21	0.0292	1.0393
	Trehalose	0.07	0.0001	4.9624
	Tryptophan	0.41	0.0095	1.3489
	Uridine 5′-monophosphate(UMP)	35.91	0.0000	1.5861
	Xanthine	0.22	0.0042	6.6433
14 d–28 d	1,3,5-benzenetriol	1.39	0.0476	1.3281
	1-Aminocyclopropanecarboxylic acid	4.03	0.0078	1.0134
	2′-deoxyuridine 5′-monophosphate	1.84	0.0075	1.0207
	2-keto-d-gluconic acid	0.18	0.0001	6.3840
	4-hydroxyphenylpyruvate	0.24	0.0107	1.9930
	Alpha-D-Glucose	3.06	0.0255	1.0604
	Alpha-ketoglutarate	0.18	0.0001	1.7002
	Carnosine	3.33	0.0100	1.0835
	Chorismic acid	11.70	0.0003	2.5018
	Cytidine 5′-diphosphocholine	1.97	0.0026	1.4115
	D-arabinonic acid	0.15	0.0158	6.6655
	D-arabinose	2.22	0.0372	1.4611
	D-glucose 6-phosphate	2.13	0.0325	1.4677
	D-glutamine	16.68	0.0074	4.3377
	D-Mannose	3.99	0.0205	6.4959
	D-mannose 6-phosphate	1.81	0.0306	4.8248
	D-ribose 5-phosphate	1.65	0.0249	2.1307
	DL-serine	3.14	0.0327	1.3572
	DL-threonine	2.39	0.0190	2.2059
	DL-tryptophan	0.57	0.0088	1.2294
	Inosine 5′-monophosphate(IMP)	3.96	0.0293	2.2679
	L-(+)-lactic acid	1.66	0.0174	17.8540
	L-Arabinono-1,4-lactone	0.21	0.0087	15.0815
	L-citrulline	0.49	0.0159	1.6141
	Malonic acid	0.22	0.0003	2.6878
	N-.alpha.-acetyl-l-ornithine	3.06	0.0257	1.2450
	N.epsilon.-acetyl-l-lysine	0.20	0.0352	1.7682
	Orotate	0.13	0.0035	1.6141
	Phenylpyruvate	0.25	0.0199	1.9329
	Pyruvaldehyde	2.76	0.0277	1.0750
	Pyruvate	0.31	0.0068	27.7142
	sn-Glycerol 3-phosphoethanolamine	1.89	0.0492	1.2950
	Succinic semialdehyde	1.31	0.0065	1.1460
	Tryptophan	0.60	0.0370	2.0075

## Data Availability

The original contributions presented in the study are included in the article and Supplementary Materials; further inquiries can be directed to the corresponding author.

## Supplementary Materials

The following supporting information can be downloaded at: https://www.mdpi.com/article/10.3390/ani16030508/s1, Figure S1: Venn Diagram of DEMs Across Different Postmortem; Figure S2: Clustered Heatmap of Differentially Expressed Metabolites (DEMs) in Enriched Pathways; Table S1: Differentially expressed metabolites in Yili horses after 0 days and 14 days of postmortem aging; Table S2: Differentially expressed metabolites in Yili horses after 14 days and 28 days of postmortem aging; Table S3: Enriched Metabolites in the Black Module.
