# Supplementary figures and images for: Dynamic Metabolic Changes During Postmortem Aging of Yili Horsemeat Revealed by Untargeted Metabolomics
Source: Animals (Basel). 2026 Feb 5;16(3):508. doi: 10.3390/ani16030508 (PMC12897160; doi:10.3390/ani16030508)

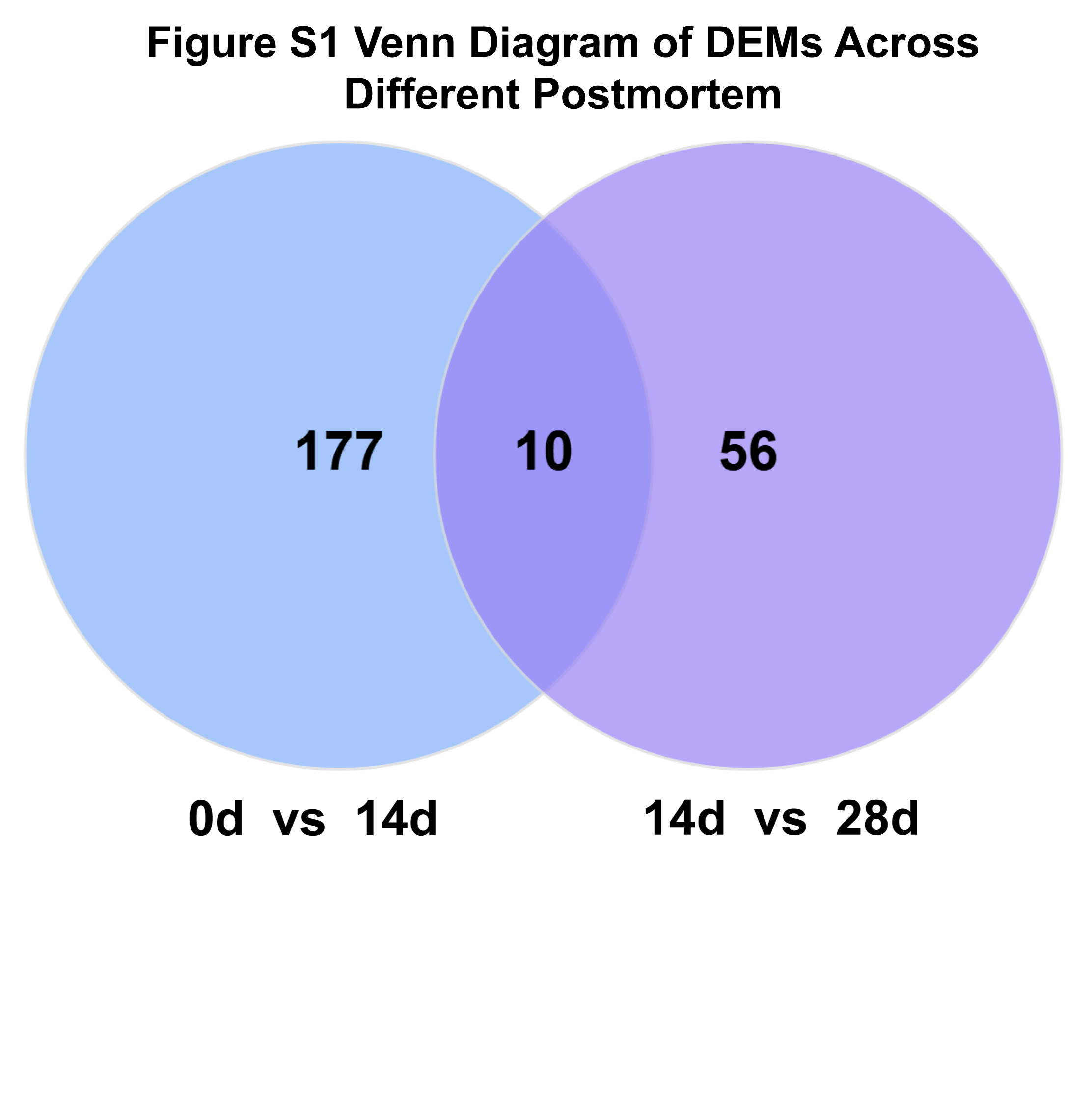

Supplement: Supplementary file 1 [file animals-16-00508-s001.zip › Supplementary Materials/Figure S1.tif]

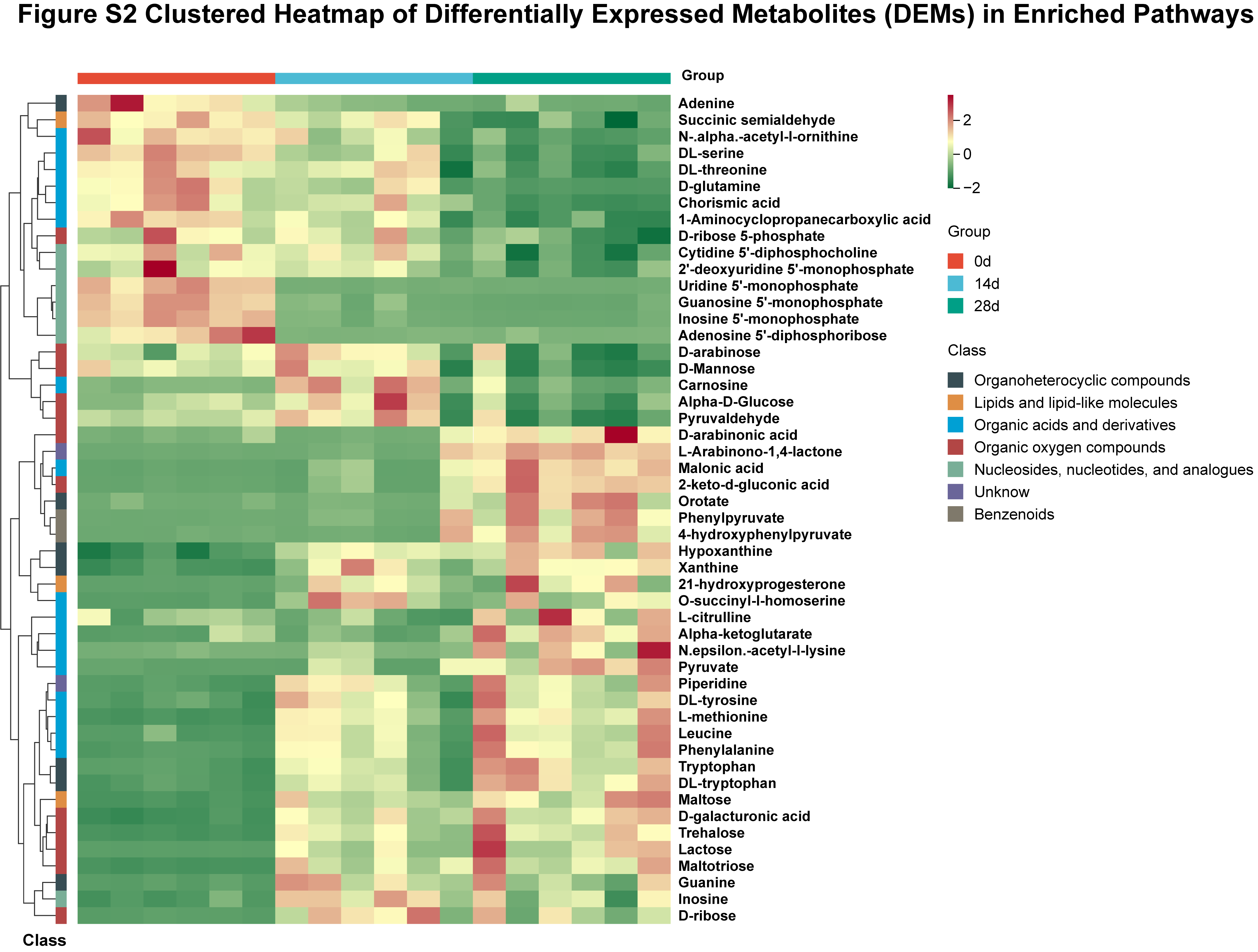

Supplement: Supplementary file 1 [file animals-16-00508-s001.zip › Supplementary Materials/Figure S2.tif]
